# 2-(2-Iodo­phen­yl)isoindoline-1,3-dione

**DOI:** 10.1107/S1600536811006544

**Published:** 2011-03-12

**Authors:** Güneş Demirtaş, Necmi Dege, Ayşen Alaman Ağar, Orhan Büyükgüngör

**Affiliations:** a Ondokuz Mayıs University, Arts and Sciences Faculty, Department of Physics, 55139 Samsun, Turkey; b Ondokuz Mayıs University, Arts and Sciences Faculty, Department of Chemistry, 55139 Samsun, Turkey

## Abstract

In the title compound, C_14_H_8_INO_2_, the dihedral angle between the isoindole ring and the phenyl ring of the 1-iodo­benzene group is 84.77 (15)°. There is a short inter­molecular I⋯O contact of 3.068 (3) Å in the crystal.

## Related literature

For the biological activity of phthalimides, see: Kerrigan *et al.* (2000 ▶); Lima *et al.* (2002 ▶). For the crystal structures of phthalimide derivatives, see: Devarajegowda *et al.* (2010 ▶); Sakthivel *et al.* (2007*a* ▶,*b* ▶); Nagaraj *et al.* (2005 ▶).
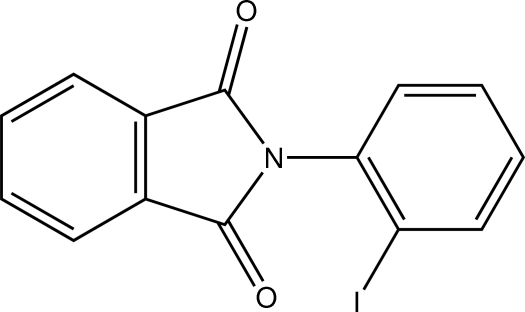


## Experimental

### 

#### Crystal data

C_14_H_8_INO_2_
*M* *_r_* = 349.11
Monoclinic, 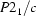

*a* = 11.5318 (5) Å
*b* = 8.0597 (2) Å
*c* = 15.6134 (7) Å
β = 118.157 (3)°
*V* = 1279.42 (9) Å^3^
*Z* = 4
Mo *K*α radiation
μ = 2.50 mm^−1^
*T* = 296 K
0.69 × 0.51 × 0.28 mm

#### Data collection

Stoe IPDS 2 diffractometer
Absorption correction: integration (*X-RED32*; Stoe & Cie, 2002 ▶) *T*_min_ = 0.291, *T*_max_ = 0.590
13075 measured reflections
2517 independent reflections
2434 reflections with *I* > 2σ(*I*)
*R*_int_ = 0.033

#### Refinement

*R*[*F* ^2^ > 2σ(*F* ^2^)] = 0.031
*wR*(*F* ^2^) = 0.078
*S* = 1.11
2517 reflections
163 parameters
H-atom parameters constrained
Δρ_max_ = 0.82 e Å^−3^
Δρ_min_ = −1.05 e Å^−3^

### 

Data collection: *X-AREA* (Stoe & Cie, 2002 ▶); cell refinement: *X-AREA*; data reduction: *X-RED32* (Stoe & Cie, 2002 ▶); program(s) used to solve structure: *WinGX* (Farrugia, 1999 ▶) and *SHELXS97* (Sheldrick, 2008 ▶); program(s) used to refine structure: *WinGX* (Farrugia, 1999 ▶) and *SHELXL97* (Sheldrick, 2008 ▶); molecular graphics: *ORTEP-3 for Windows* (Farrugia, 1997 ▶); software used to prepare material for publication: *WinGX* and *PLATON* (Spek, 2009 ▶).

## Supplementary Material

Crystal structure: contains datablocks I, global. DOI: 10.1107/S1600536811006544/lw2057sup1.cif

Structure factors: contains datablocks I. DOI: 10.1107/S1600536811006544/lw2057Isup2.hkl

Additional supplementary materials:  crystallographic information; 3D view; checkCIF report

## Figures and Tables

**Fig. 1.**
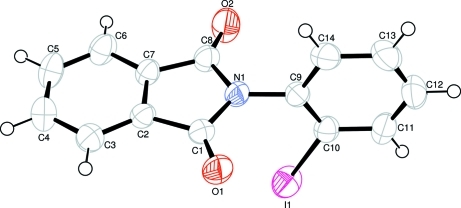
The asymmetric unit of the title compound, showing the atomic numbering scheme. Displacement ellipsoids are drawn at the 50% probability level.

**Fig. 2.**
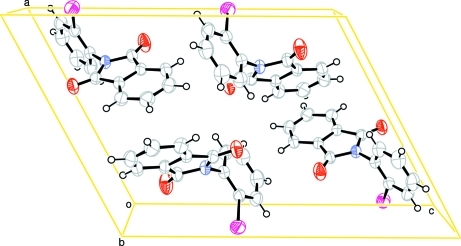
The crystal packing of the title compound in the unit cell.

## supplementary crystallographic information

### Comment

The importance of the biological activity of the phthalimide group with reference to *N*-2-Iodophenylphthalimide is described by Kerrigan *et al.*, (2000) and Lima *et al.*, (2002).

The C—C bond distances are 1.485 (4) Å for C1—C2 and 1.481 (4)Å for C7—C8. The bond distances between carbon atoms in the aromatic rings range from 1.369 (6)Å to 1.390 (5)Å. The C1=O1 and C8=O2 bond distances are 1.204 (3)Å and 1.196 (4)Å, respectively. The N—C bond distances range from 1.399 (4) Å to 1.432 (4)Å. These values are consistent with those reported in the literature (Devarajegowda *et al.*, 2010; Sakthivel *et al.*, 2007*a*; Sakthivel *et al.*2007*b*; Nagaraj *et al.*, 2005). C10—I1 bond distance is 2.094 (3) Å. An intermolecular I1···O2 contact of 3.068 (3)Å is present in the crystal.

### Experimental

The compound *N*-2-Iodophenylphthalimide was prepared by refluxing a mixture of a solution containing *N*-hydroxyphthalimide (0.0113 g 0.069 mmol) in 20 ml ethanol and a solution containing 4-amino-4-methylphenol (0.0303 g 0.069 mmol) in 20 ml ethanol. The reaction mixture was stirred for 1h under reflux. The crystals of *N*-2-Iodophenylphthalimide suitable for X-ray analysis were obtained from ethyl alcohol by slow evaporation (yield % 41; m.p 180.3–184.0 *^o^*C).

### Refinement

All hydrogen atoms were positioned geometrically (C—H=0.93 Å) and treated as riding with *U*_iso_(H)=1.1*U*_eq_(C).

### Crystal data

**Table:**

C_14_H_8_INO_2_	*F*(000) = 672
*M**_r_* = 349.11	*D*_x_ = 1.812 Mg m^−^^3^
Monoclinic, *P*2_1_/*c*	Melting point: 455 K
Hall symbol: -P 2ybc	Mo *K*α radiation, λ = 0.71073 Å
*a* = 11.5318 (5) Å	Cell parameters from 26549 reflections
*b* = 8.0597 (2) Å	θ = 1.8–28.0°
*c* = 15.6134 (7) Å	µ = 2.50 mm^−^^1^
β = 118.157 (3)°	*T* = 296 K
*V* = 1279.42 (9) Å^3^	Prism, red
*Z* = 4	0.69 × 0.51 × 0.28 mm

### Data collection

**Table:**

Stoe IPDS 2 diffractometer	2517 independent reflections
Radiation source: fine-focus sealed tube	2434 reflections with *I* > 2σ(*I*)
plane graphite	*R*_int_ = 0.033
rotation method scans	θ_max_ = 26.0°, θ_min_ = 2.0°
Absorption correction: integration (*X-RED32*; Stoe & Cie, 2002)	*h* = −14→14
*T*_min_ = 0.291, *T*_max_ = 0.590	*k* = −9→9
13075 measured reflections	*l* = −19→19

### Refinement

**Table:**

Refinement on *F*^2^	Primary atom site location: structure-invariant direct methods
Least-squares matrix: full	Secondary atom site location: difference Fourier map
*R*[*F*^2^ > 2σ(*F*^2^)] = 0.031	Hydrogen site location: inferred from neighbouring sites
*wR*(*F*^2^) = 0.078	H-atom parameters constrained
*S* = 1.11	*w* = 1/[σ^2^(*F*_o_^2^) + (0.0373*P*)^2^ + 1.3378*P*] where *P* = (*F*_o_^2^ + 2*F*_c_^2^)/3
2517 reflections	(Δ/σ)_max_ = 0.002
163 parameters	Δρ_max_ = 0.82 e Å^−^^3^
0 restraints	Δρ_min_ = −1.05 e Å^−^^3^

### Special details

**Table:**

Geometry. All e.s.d.'s (except the e.s.d. in the dihedral angle between two l.s. planes) are estimated using the full covariance matrix. The cell e.s.d.'s are taken into account individually in the estimation of e.s.d.'s in distances, angles and torsion angles; correlations between e.s.d.'s in cell parameters are only used when they are defined by crystal symmetry. An approximate (isotropic) treatment of cell e.s.d.'s is used for estimating e.s.d.'s involving l.s. planes.
Refinement. Refinement of *F*^2^ against ALL reflections. The weighted *R*-factor *wR* and goodness of fit *S* are based on *F*^2^, conventional *R*-factors *R* are based on *F*, with *F* set to zero for negative *F*^2^. The threshold expression of *F*^2^ > σ(*F*^2^) is used only for calculating *R*-factors(gt) *etc*. and is not relevant to the choice of reflections for refinement. *R*-factors based on *F*^2^ are statistically about twice as large as those based on *F*, and *R*- factors based on ALL data will be even larger.

### Fractional atomic coordinates and isotropic or equivalent isotropic displacement parameters (Å^2^)

**Table:**

	*x*	*y*	*z*	*U*_iso_*/*U*_eq_	
C1	0.6644 (3)	0.2407 (4)	0.1170 (2)	0.0391 (6)	
C2	0.6487 (3)	0.3409 (4)	0.1906 (2)	0.0388 (6)	
C3	0.5850 (3)	0.4891 (4)	0.1819 (2)	0.0500 (7)	
H3	0.5418	0.5438	0.1225	0.060*	
C4	0.5880 (4)	0.5537 (5)	0.2652 (3)	0.0581 (8)	
H4	0.5485	0.6557	0.2622	0.070*	
C5	0.6482 (4)	0.4699 (5)	0.3524 (3)	0.0588 (9)	
H5	0.6469	0.5156	0.4066	0.071*	
C6	0.7105 (4)	0.3194 (4)	0.3610 (2)	0.0535 (8)	
H6	0.7506	0.2624	0.4197	0.064*	
C7	0.7105 (3)	0.2578 (4)	0.2786 (2)	0.0405 (6)	
C8	0.7669 (3)	0.1017 (4)	0.2642 (2)	0.0445 (6)	
C9	0.7720 (3)	−0.0319 (3)	0.12144 (19)	0.0392 (6)	
C10	0.8848 (3)	−0.0215 (4)	0.1120 (2)	0.0425 (6)	
C11	0.9183 (4)	−0.1504 (5)	0.0691 (2)	0.0540 (8)	
H11	0.9939	−0.1433	0.0621	0.065*	
C12	0.8393 (5)	−0.2892 (4)	0.0369 (3)	0.0630 (10)	
H12	0.8621	−0.3762	0.0087	0.076*	
C13	0.7274 (5)	−0.2994 (4)	0.0464 (3)	0.0627 (10)	
H13	0.6744	−0.3933	0.0243	0.075*	
C14	0.6924 (4)	−0.1705 (4)	0.0887 (3)	0.0527 (8)	
H14	0.6162	−0.1774	0.0949	0.063*	
I1	1.00579 (2)	0.18859 (3)	0.158933 (17)	0.06096 (11)	
N1	0.7345 (2)	0.0987 (3)	0.16550 (16)	0.0399 (5)	
O1	0.6253 (2)	0.2689 (3)	0.03201 (15)	0.0534 (5)	
O2	0.8266 (3)	−0.0042 (3)	0.32190 (18)	0.0722 (8)	

### Atomic displacement parameters (Å^2^)

**Table:**

	*U*^11^	*U*^22^	*U*^33^	*U*^12^	*U*^13^	*U*^23^
C1	0.0420 (14)	0.0404 (14)	0.0373 (14)	0.0006 (12)	0.0207 (12)	0.0023 (12)
C2	0.0407 (14)	0.0423 (14)	0.0353 (13)	0.0008 (11)	0.0196 (12)	0.0009 (11)
C3	0.0539 (17)	0.0491 (17)	0.0505 (16)	0.0086 (14)	0.0277 (14)	0.0063 (14)
C4	0.060 (2)	0.0550 (19)	0.065 (2)	0.0098 (16)	0.0335 (17)	−0.0071 (16)
C5	0.0586 (19)	0.071 (2)	0.0498 (17)	0.0052 (17)	0.0283 (16)	−0.0170 (16)
C6	0.0536 (18)	0.070 (2)	0.0362 (15)	0.0082 (15)	0.0204 (14)	−0.0010 (14)
C7	0.0411 (14)	0.0462 (15)	0.0354 (13)	0.0040 (12)	0.0192 (12)	0.0000 (12)
C8	0.0501 (16)	0.0492 (17)	0.0353 (13)	0.0087 (13)	0.0211 (12)	0.0053 (12)
C9	0.0462 (15)	0.0376 (14)	0.0340 (13)	0.0001 (11)	0.0191 (12)	−0.0003 (11)
C10	0.0453 (15)	0.0443 (15)	0.0374 (14)	−0.0020 (12)	0.0191 (12)	−0.0035 (12)
C11	0.0600 (19)	0.0583 (19)	0.0499 (17)	0.0068 (16)	0.0309 (16)	−0.0063 (15)
C12	0.089 (3)	0.0476 (18)	0.058 (2)	0.0041 (17)	0.039 (2)	−0.0110 (15)
C13	0.087 (3)	0.0417 (17)	0.061 (2)	−0.0143 (17)	0.037 (2)	−0.0098 (15)
C14	0.064 (2)	0.0464 (17)	0.0532 (18)	−0.0091 (14)	0.0324 (16)	−0.0014 (14)
I1	0.05713 (16)	0.06921 (18)	0.05964 (17)	−0.02284 (10)	0.03011 (12)	−0.02193 (10)
N1	0.0480 (13)	0.0408 (12)	0.0341 (11)	0.0045 (10)	0.0222 (10)	0.0021 (9)
O1	0.0687 (15)	0.0578 (13)	0.0354 (11)	0.0109 (11)	0.0259 (11)	0.0079 (10)
O2	0.102 (2)	0.0705 (16)	0.0473 (13)	0.0420 (16)	0.0381 (14)	0.0217 (12)

### Geometric parameters (Å, °)

**Table:**

C1—O1	1.204 (3)	C8—O2	1.196 (4)
C1—N1	1.399 (4)	C8—N1	1.403 (3)
C1—C2	1.485 (4)	C9—C10	1.379 (4)
C2—C3	1.374 (4)	C9—C14	1.382 (4)
C2—C7	1.385 (4)	C9—N1	1.432 (4)
C3—C4	1.387 (5)	C10—C11	1.385 (4)
C3—H3	0.9300	C10—I1	2.094 (3)
C4—C5	1.377 (5)	C11—C12	1.379 (5)
C4—H4	0.9300	C11—H11	0.9300
C5—C6	1.384 (5)	C12—C13	1.369 (6)
C5—H5	0.9300	C12—H12	0.9300
C6—C7	1.379 (4)	C13—C14	1.390 (5)
C6—H6	0.9300	C13—H13	0.9300
C7—C8	1.481 (4)	C14—H14	0.9300
			
O1—C1—N1	124.8 (3)	N1—C8—C7	105.8 (2)
O1—C1—C2	129.2 (3)	C10—C9—C14	120.4 (3)
N1—C1—C2	106.0 (2)	C10—C9—N1	121.4 (3)
C3—C2—C7	121.3 (3)	C14—C9—N1	118.2 (3)
C3—C2—C1	130.5 (3)	C9—C10—C11	119.9 (3)
C7—C2—C1	108.1 (2)	C9—C10—I1	121.2 (2)
C2—C3—C4	117.2 (3)	C11—C10—I1	118.9 (2)
C2—C3—H3	121.4	C12—C11—C10	119.8 (3)
C4—C3—H3	121.4	C12—C11—H11	120.1
C5—C4—C3	121.4 (3)	C10—C11—H11	120.1
C5—C4—H4	119.3	C13—C12—C11	120.2 (3)
C3—C4—H4	119.3	C13—C12—H12	119.9
C4—C5—C6	121.5 (3)	C11—C12—H12	119.9
C4—C5—H5	119.3	C12—C13—C14	120.4 (3)
C6—C5—H5	119.3	C12—C13—H13	119.8
C7—C6—C5	117.0 (3)	C14—C13—H13	119.8
C7—C6—H6	121.5	C9—C14—C13	119.2 (3)
C5—C6—H6	121.5	C9—C14—H14	120.4
C6—C7—C2	121.6 (3)	C13—C14—H14	120.4
C6—C7—C8	129.9 (3)	C1—N1—C8	111.5 (2)
C2—C7—C8	108.5 (2)	C1—N1—C9	124.7 (2)
O2—C8—N1	125.1 (3)	C8—N1—C9	123.7 (2)
O2—C8—C7	129.0 (3)		
			
O1—C1—C2—C3	1.0 (6)	C14—C9—C10—I1	179.0 (2)
N1—C1—C2—C3	−178.1 (3)	N1—C9—C10—I1	−1.4 (4)
O1—C1—C2—C7	180.0 (3)	C9—C10—C11—C12	−0.6 (5)
N1—C1—C2—C7	0.9 (3)	I1—C10—C11—C12	−179.4 (3)
C7—C2—C3—C4	1.5 (5)	C10—C11—C12—C13	0.6 (6)
C1—C2—C3—C4	−179.7 (3)	C11—C12—C13—C14	−0.2 (6)
C2—C3—C4—C5	−2.2 (5)	C10—C9—C14—C13	0.1 (5)
C3—C4—C5—C6	1.3 (6)	N1—C9—C14—C13	−179.5 (3)
C4—C5—C6—C7	0.5 (6)	C12—C13—C14—C9	−0.2 (6)
C5—C6—C7—C2	−1.2 (5)	O1—C1—N1—C8	179.7 (3)
C5—C6—C7—C8	−179.4 (3)	C2—C1—N1—C8	−1.2 (3)
C3—C2—C7—C6	0.3 (5)	O1—C1—N1—C9	1.3 (5)
C1—C2—C7—C6	−178.8 (3)	C2—C1—N1—C9	−179.6 (3)
C3—C2—C7—C8	178.8 (3)	O2—C8—N1—C1	−180.0 (3)
C1—C2—C7—C8	−0.3 (3)	C7—C8—N1—C1	1.0 (3)
C6—C7—C8—O2	−1.0 (6)	O2—C8—N1—C9	−1.5 (5)
C2—C7—C8—O2	−179.4 (4)	C7—C8—N1—C9	179.4 (3)
C6—C7—C8—N1	178.0 (3)	C10—C9—N1—C1	84.2 (4)
C2—C7—C8—N1	−0.4 (3)	C14—C9—N1—C1	−96.2 (3)
C14—C9—C10—C11	0.3 (4)	C10—C9—N1—C8	−94.1 (4)
N1—C9—C10—C11	179.9 (3)	C14—C9—N1—C8	85.6 (4)
